# Progress and challenges in exploring aquatic microbial communities using non-targeted metabolomics

**DOI:** 10.1038/s41396-023-01532-8

**Published:** 2023-10-19

**Authors:** Monica Thukral, Andrew E. Allen, Daniel Petras

**Affiliations:** 1 University of California San Diego, Scripps Institution of Oceanography, La Jolla, CA USA; 2 J. Craig Venter Institute, Microbial and Environmental Genomics Group, La Jolla, CA USA; 3 University of Tuebingen, CMFI Cluster of Excellence, Tuebingen, Germany; 4 University of California Riverside, Department of Biochemistry, Riverside, CA USA

**Keywords:** Microbial ecology, Microbial ecology

## Abstract

Advances in bioanalytical technologies are constantly expanding our insights into complex ecosystems. Here, we highlight strategies and applications that make use of non-targeted metabolomics methods in aquatic chemical ecology research and discuss opportunities and remaining challenges of mass spectrometry-based methods to broaden our understanding of environmental systems.

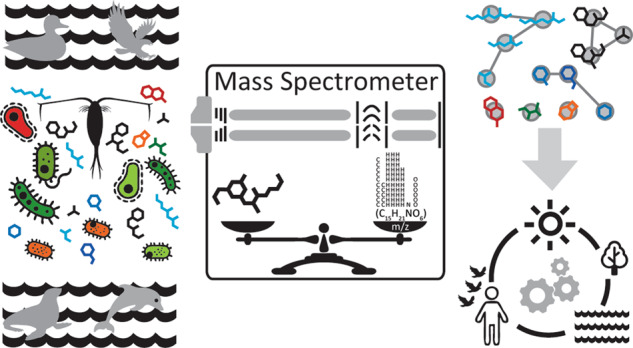

## Introduction

The field of aquatic microbiology has been very successfull in implementing and using molecular biological techniques such as nucleic acid sequencing and cloning/conjugation to study individual microorganisms and ecosystem function [1, 2]. In the last decades, tools used in the field of molecular biology have constantly evolved and expanded, including emerging omics tools such as transcriptomics, genomics, and proteomics [3]. While there are conflicting viewpoints on whether molecular biology is defined by the level of life at which biology is studied, omics tools have been revolutionary to studying biology on the molecular level and are now widely used in many molecular biology studies [4–6].

**Fig. 1:**
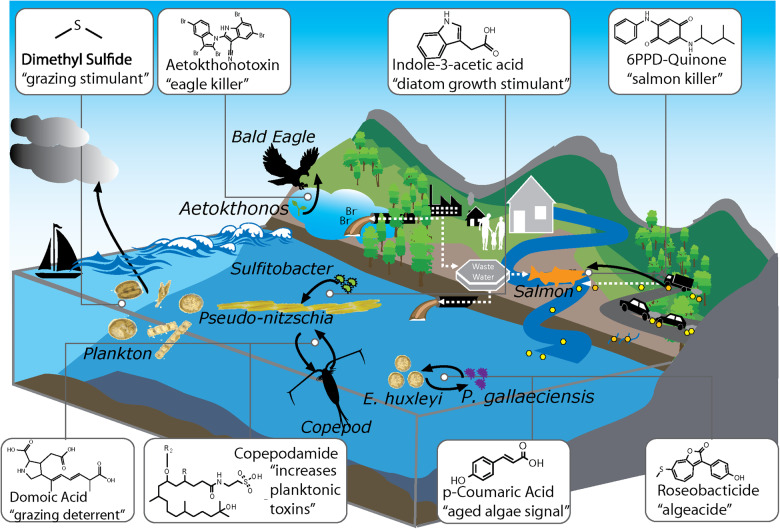
Small molecules mediate microbial community function. Depicted includes photosynthetic microalgae producing dimethyl sulfide inducing both cloud formation and grazing by zooplankton, and on the other hand *Pseudo-nitzschia*’s production of domoic acid as a grazing deterrent. In addition, *Sulfitobacter’s* production of indole-3-acetic acid increases planktonic toxins like domoic acid from *Pseudo-nitzschia*. Furthermore, when *an E. huxleyi* bloom ages, it produces p-coumaric acid, with response to which *P. gallaeciensis* begins to produce algaecide roseobacticide. In freshwater nearby, *Aetokthonos* cyanobacteria colonizes a freshwater invasive plant *Hydrilla* and produces aetokthonotoxin which bioaccumulates up the food web to top predator bald eagles, causing neurological disease. On a road nearby, cars’ rubber tires contain 6PPD which runs off into freshwater creeks and is transformed to 6PPD-quinone, causing salmon mortality.

Mass spectrometry (MS)-based metabolomics methods, both targeted and non-targeted, are becoming widely used tools to study aquatic ecosystems [7–9]. Targeted metabolomics typically refers to detecting and quantifying a specific metabolite or set of metabolites [10, 11]. Non-targeted metabolomics on the other hand, aims to detect all metabolites within a sample, typically used as a discovery approach to generate hypotheses about the identity, origin, function and effects of small molecules in biological systems. The study design, including whether to use targeted or non-targeted metabolomics depends on whether the driving question and nature of the study is to investigate already known molecules or pathways, or whether it is to characterize the total chemical diversity of a biological system. Natural product chemistry studies on the other hand have a long-standing tradition of isolating organic compounds from isolated organisms or biomass and elucidating their structures with multi-modal approaches such as MS and nuclear magnetic resonance (NMR) spectroscopy and assess their biological activity, mainly in the context of pharmaceutical properties [12, 13]. While these methods inform organismal presence/absence as well as molecular insights and functionalities, the field has only scratched the surface of understanding chemical exchange in complex microbial community dynamics [14, 15]. Although there has been great progress in microbial and chemical ecology studies, with many new molecular insights into different ecosystems, we are far from fully capturing and understanding how microorganisms affect each other through the multitude of other metabolites they produce (Fig. 1).

Non-targeted metabolomics promises to provide information of which molecules are present, exchanged, and modified in a given (eco)system, which may illuminate the black box of organismal and community metabolomes. The field of lipidomics is often considered to be a specialized sub-field of metabolomics, and both targeted and non-targeted lipidomics methods have contributed significantly to our understanding of aquatic metabolomes [16, 17]. Over the past 30 years, significant advances have been made in the fields of non-targeted metabolomics as well as nucleotide sequencing. Within the last years, long-read-sequencing has emerged as new sequencing strategy, which are particularly useful for the assembly of metagenomes [18], that include repetitive gene sequences in mega-synthetases of specialized metabolites, such as non-ribosomal peptide synthetases (NRPS, e.g. Microcystins) or polyketide synthetases (PKS, e.g. Brevetoxin) [19, 20].

**Fig. 2:**
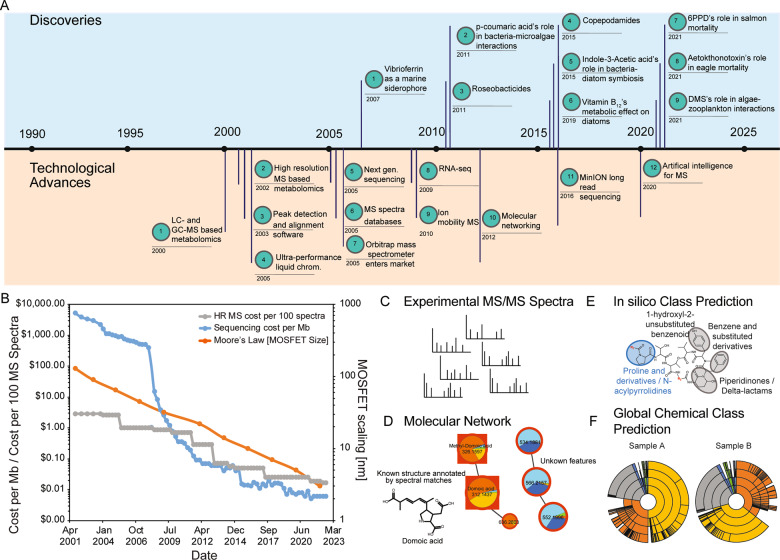
Technological advances drive discoveries in Harmful algae bloom research. Panel (**A**) shows a timeline of, in our opinion, important discoveries of metabolites and/or their roles in aquatic ecosystem function as well as technological advances that were made and will be important for new molecular insights. Panel (**B**) shows the development of cost of MS and sequencing analysis in comparison to Moore’s law shown as logarithmic decrease of transistor size (MOSFET scaling), updated from previous work [160] and assuming 25€/h instrument run time according to Deutsche Forschungsgemeinschaft (DFG) Guidelines for Instrumentation Usage Costs and Core Facilities. Panels **C** through **F** show examples of new metabolite data annotation and visualization methods through molecular networks and class level annotation of metabolites [47, 109, 115]. Panel **D** shows an example of a GNPS molecular network of metabolomes from Pseudo-nitzschia cultures that annotated domoic acid by spectral matches in addition to unknown features [47]. Panel **E** shows rivulariapeptolide [115], for which structures were annotated using new tools like CANOPUS [109].

Technological advances in nucleic acid sequencing and in mass spectrometry have occurred concurrently and are both key to enabling chemical ecological discoveries (Fig. 2A). Gas Chromatography- Mass Spectrometry (GC-MS) was developed in the late 50 s and has been widely used to identify and quantify metabolites [21–24]. LC- and GC-MS-based metabolomics, high-resolution MS-based metabolomics, peak detection and alignment software, and ultra-performance liquid chromatography became widely accessible in the early 2000s [25–28]. In the mid 2000s, the orbitrap mass spectrometer entered the market and the first tandem mass spectrometry databases were developed, concurrent with the introduction of next generation sequencing [29–33]. These advances were followed by RNA-seq and ion mobility mass spectrometry by 2009 [34, 35]. Over the last decade, sensitivity, resolution, and scan speed of both orbitrap and Q-ToF-based platforms have significantly increased [36]. Other recent advances include molecular networking and long read sequencing, and finally the contemporary revolution of machine learning tools and artificial intelligence [37–42].

In parallel to the decreasing cost of nucleic acid sequencing, the cost of acquiring an equivalent amount of MS data has significantly decreased as well (Fig. 2B). However, despite the technological advances, decreasing costs, and wider accessibility of metabolomics tools, major bottlenecks remain. For example, currently only 5–10% of spectra in most non-targeted metabolomics experiments can be annotated as known molecules: the remaining 90–95% of spectra are unknown and considered as “dark matter” which contains vast unknown knowledge space [14]. In addition to providing examples of the successful use of metabolomics tools to study aquatic ecosystems, we will discuss key bottle necks and emerging solutions in this article.

## Success stories of deciphering chemical mediators in aquatic chemical ecology

### Metabolomics tools enable the study of community dynamics of bloom-forming organisms

**Fig. 3:**
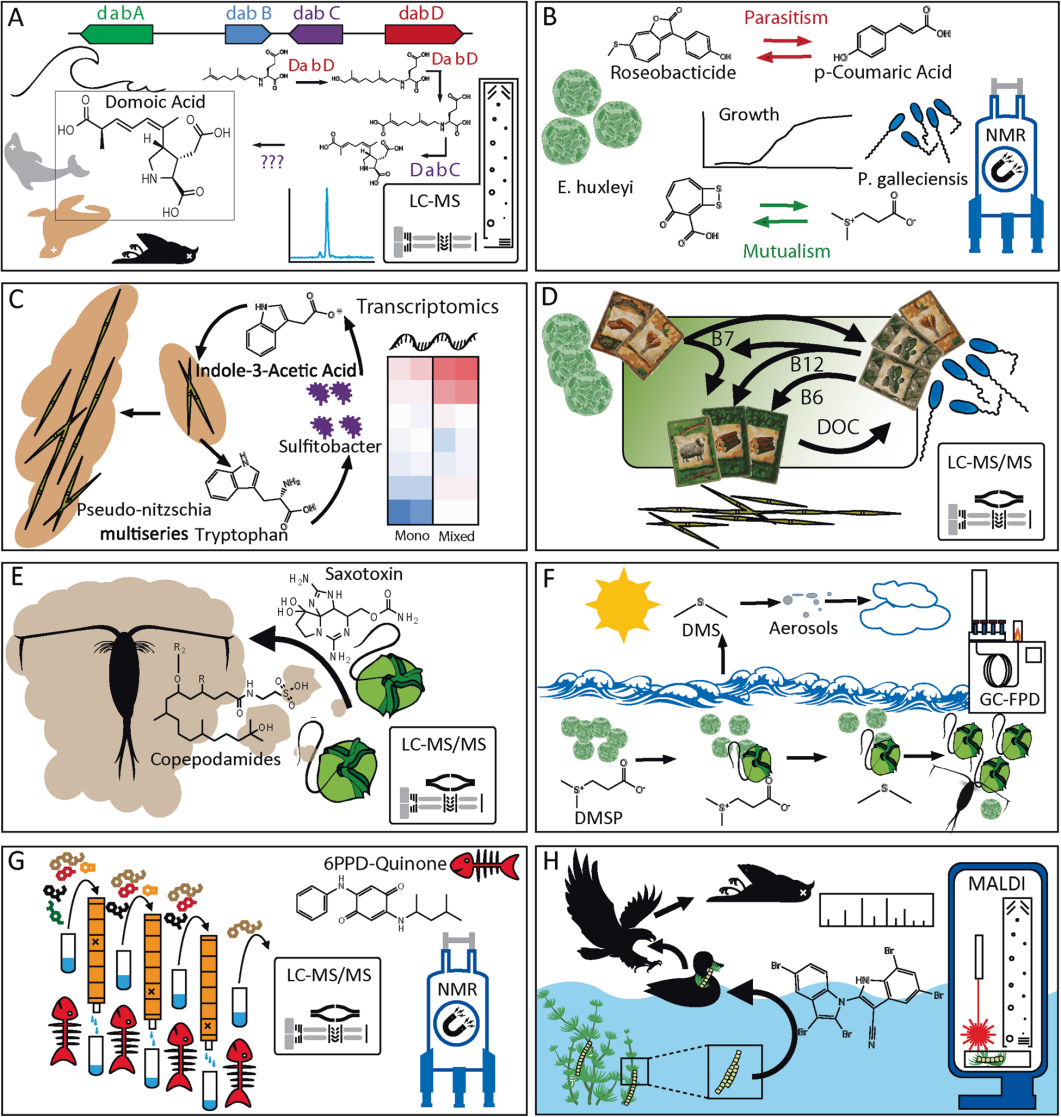
Success stories of deciphering chemical mediators in in aquatic chemical ecology. Domoic acid’s biosynthetic pathway was characterized with transcriptomics, biochemistry and NMR (**a**), p-coumaric acid and roseobacticide are exchanged through a parasitic interaction between a phytoplankton and a bacteria characterized through dose-response growth experiments and NMR (**b**), and metabolomics and transcriptomics were used to characterize the exchange of indole-3-acetic acid and tryptophan between a diatom and symbiotic bacteria (**c**). Many microorganisms have not retained the ability to biosynthesize cobalamin (Vitamin B_12_) and rely on other microorganisms to produce it in exchange for other resources (**d**). Copepodamides were the first molecules discovered to mediate “chemical warfare” between zooplankton and their prey (**e**), and DMS has been known to modulate cloud formation and climate and a new role has been discovered to modulate algae-grazer interactions through GC-FPD (**f**), 6PPD from tire runoff into water sources and its ecological effect was characterized UPLC-HRMS/MS and NMR (**g**), and AP-MALDI-MSI was used to study cyanobacteria colonies on plant leaves to detect aetokthonotoxin (**h**).

Metabolomics techniques have rapidly enhanced the study of cosmopolitan bloom-forming organisms: cyanobacteria, diatoms, dinoflagellates and haptophytes (Table 1, Fig. 1, Fig. 3). Targeted metabolomics has been used to determine how commonly used algaecides affect toxin production of cyanobacteria *Microcystis aeruginosa* microcystins [43]. Here, targeted metabolomics led to the discovery of how algaecides alter the total metabolome. For example, exogenous addition of copper sulfate decreased metabolites associated with oxidative stress, whereas hydrogen peroxide and sodium carbonate peroxidase increased those oxidative stress metabolites. This work established fundamental data that can be leveraged by policy-makers in deciding how to treat *Microcystis* blooms while managing toxin production. In addition to benefiting policymakers addressing cyanobacterial blooms through targeted metabolomics, non-targeted metabolomics has been used in cyanobacterial research to discover new compounds.

**Table 1:** Examples of chemical cue characterization over time.

Molecule or discovery	Functional level (ecosystem, lab mesocosm, organism, in vitro)	Year of characterization	Select techniques in characterization
Smenamide A and B, and Smenthiazole A as cytotoins in a *Trichodesmium* bloom [44]	Ecosystem	2018	LC-MS/MS, NMR.
Domoic Acid Biosynthesis [45]	In Vitro	2018	Transcriptomics, MS, NMR.
Azelaic Acid and Rosmarinic Acid’s role in a diatom modulating bacterial community [60]	Lab mesocosm	2020	Transcriptomics, MS.
Roseobacticides [61]	In Vitro, Lab Mesocosm	2011	NMR, LC-MS.
p-coumaric Acid’s role in bacterial-microalgal interactions [62]	In Vitro, Lab Mesocosm	2011	NMR, LC-MS.
Indole-3-Acetic Acid’s role in symbiosis between bacteria and diatoms [66]	Lab Mesocosm.	2015	Growth experiments, Genomics, Transcriptomics, MS.
Vitamin B12’s metabolic effect on diatoms [70]	Ecosystem, Lab mesocosm	2019	Growth Experiments, MS.
Vibrioferrin as a marine siderophore [73]	In Vitro	2007	HPLC, NMR, MS.
Copepodamides [79]	Ecosystem, Lab mesocosm	2015	Degradative chemical experiments, NMR, MS.
DMS’s role in algae-zooplankton interactions [84]	Ecosystem, Lab Mesocosm	2021	Genetic Transformation, GC-FPD, Growth experiments.
6PPD’s role in salmon mortality [89]	Ecosystem, Lab mesocosm	2021	LC-MS/MS, NMR.
Aetokthonotoxin’s role in eagle mortality [90]	Ecosystem, In Vitro Gene/Protein	2021	AP-MALDI-MSI, LC-MS/MS, NMR, Crystallography, Imaging,
Rivulariapeptolides as serine protease inhibitors [115]	In Vitro	2022	Native metabolomics, MS, NMR.

Thirteen discoveries, alongside the functional level of the molecule or discovery, the year of characterization, and selected techniques used in the characterization.

For example, when the cyanobacterial genus *Trichodesmium*, an open-ocean bloom-forming organism that fixes nitrogen gas into organic nitrogen was interrogated with non-targeted metabolomics using MS/MS-based molecular networking, three cytotoxic compounds were discovered in the bloom: smenamide A, smenamide B, and smenothiazole A [44]. The discovery of these novel cytotoxic compounds by using non-targeted metabolomics allows for further probing of the ecological roles of these molecules in blooms in addition to a wealth of novel data of secondary metabolites of understudied yet globally abundant organisms such as *Trichodesmium*.

Diatom microbial ecology studies too have benefited from metabolomics methods. The genes and enzymes involved in domoic acid’s biosynthetic pathway were identified using methods including transcriptomics, MS, and NMR (Fig. 3a) [45]. The *Pseudo-nitzschia* genus is well studied due to its capability to produce the domoic acid neurotoxin which can cause mass mortality to mammals and fish in addition to significant economic downturns in coastal regions dependent on fisheries. This genus only exhibits haploid gametes during the understudied sexual phase when it encounters another cell of the opposite mating type [46]. Non-targeted metabolomics on each of the two mating types of *Pseudo*-*nitzschia multistriata* in addition to mixed mating types revealed characteristic metabolites of mating types: higher levels of the fatty acid oleamide in the mixed culture and higher levels of the bacterial osmoregulation-compound ectoine in the MT- mating type culture. Recent work discovered distinct metabolomes and microbiomes among species of *Pseudo-nitzschia* [47]. These conclusions are bases for future work studying the economically and ecologically relevant diatom genus *Pseudo*-*nitzschia*, and can support ongoing work of sexual reproduction of *Pseudo*-*nitzschia* in the laboratory.

Allelopathy, or production of small molecules that disrupt growth or kill competing species is a common competitive regulatory mechanism within aquatic microbial communities. *Karenia brevis* is a dinoflagellate that exhibits allelopathy, disrupting competing phytoplankton such as the diatoms *Asterionellopsis glacialis* and *Thalassiosira pseudonana* [48]. MS was used to elucidate the metabolic pathways involved in the dinoflagellate’s allelopathy and in the resistance of the affected phytoplankton using metabolomics and proteomics. Exposure to allelopathic molecules impacts the evolution of resistance: *A. glacialis* was more metabolically robust and exhibited higher resistance to exposure than *T. pseudonana* did, likely because of regular exposure to *K. brevis* blooms. In the sensitive, less “immune” *T. pseudonana*, exposure affected several metabolic pathways, leading to decreased photosynthetic capacity, reduced ability to osmoregulate, suppression of lipid synthesis, and increased cellular oxidative stress [48, 49]. Supplementing this conclusion, the allelopathic capacity of the exudates of five strains of *K. brevis* on *A. glacialis* were interrogated, determining that higher concentrations of uncharacterized fatty acid-derived lipids and aromatic/polyunsaturated compounds led to higher allelopathy of a strain [50]. These studies reveal the utility of metabolomics to gain a snapshot of the effects of chemical allelopathic effects of one microorganism on another, revealing dynamics and predictions of microbial community structure. Predicting how microorganisms will interact chemically is especially important as global change leads to encounters of species not historically exposed to one another.

In addition to characterizing dynamics of ecologically-relevant organisms, metabolomics has recently been utilized to typify dissolved organic matter (DOM) in the environment that the microorganisms inhabit [51]. Building upon stoichiometric ratios that are traditionally used to characterize organic matter in rivers and streams, non-targeted metabolomics was utilized to identify higher levels of flavonoids along a creek as it moved downstream. Another study described patterns and differences in river surface water metabolomes of various sizes and ecosystems using a community-science approach to amalgamate global data [52]. This data is important to understanding the sources and sinks of carbon transport, and to characterizing spatial and temporal dynamics of metabolites in the environment that microorganisms and higher-trophic organisms experience, consume, and produce.

### Metabolic “Hot Spots” reveal algae-bacteria interactions

To understand ecosystem dynamics, the phycosphere has been explored in both fresh water and marine aquatic environments through multiple-omics methods, revealing how bacterial consortia interacts with bloom-forming microalgae. The physcophere describes the region directly surrounding the algal cell in which algal extracellular exudates support bacterial growth .

Dissolved organic molecules diffusing from the algal phycosphere attract heterotrophic bacteria, and bacteria benefit from these organic molecules in exchange for cofactors that the microalgae depend on for their growth. Metabolites are exchanged at the phycosphere, and act as signaling compounds to communicate between phytoplankton and bacteria, whether in a mutualistic, antagonistic, or parasitic manner [53]. Cirri and Pohnert proposed the concept of “metabolic hotspots” around algal cells, where resource exchange occurs, and which bacteria have evolved to utilize for their metabolic needs [54]. In both terrestrial and marine aquatic environments, bacteria that inhabit algal phycospheres are not highly abundant outside that region, reflecting their specialization to inhabit the phycosphere niche [53, 55–57]. Non-targeted metabolomics breaks free from examining single microorganism-molecule interactions, and rather captures the broad chemical dynamics in the total community.

For example, heterotrophic bacteria that grow in the physcosphere of freshwater cyanobacteria *Microcystis aeruginosa* and on its DOM drew down more dissolved organic carbon than non-phycosphere-inhabiting-heterotrophs, particularly removing more lipids, organic acids and organoheterocyclic molecules [58]. The phycosphere-heterotrophs also had larger genome sizes, reflecting the wider resource pool of DOM available in the phycosphere to maintain larger genomes [59].

Diatoms have some control over their microbiome: they attract beneficial bacteria and dispel the harmful ones through unknown cellular mechanisms and metabolic signaling pathways [60]. They change their transcriptional activity when they encounter certain microbial communities, and as a result release central and secondary metabolites. In response, bacteria that are attuned to the diatom’s exudate respond transcriptionally to these metabolites with varied speeds and intensities and may consume these central metabolites. For example, azelaic acid and rosmarinic acid produced by diatom *Asterionellopsis glacialis* were found to modulate bacterial behavior and growth to simultaneously promote diatom symbionts and demote diatom opportunists [60].

In addition to symbiosis, bacteria have been shown to act as pathogens of microalgae. Roseobacter *P. gallaeciensis* blooms along blooming haptophyte *Emeliania huxleyi*. When an *E. huxleyi* bloom ages, it produces p-coumaric acid, with response to which *P. gallaeciensis* begins to produce potent algaecides called roseobacticides (Fig. 3b) [61, 62]. This dynamic interaction of the algae and bacteria encompasses mutualism and parasitism as the blooms boom and bust. P-coumaric acid and other phenolic acids, are common plant metabolites that can be released in plant exudates as well as through the breakdown of lignin. Hence, there is the possibility that *P. gallaeciensis* could also respond metabolically to p-coumaric acid that is not from an *E. huxleyi* source, but rather derived from another non-haptophyte algae or from runoff of plant-derived dissolved organic matter [63, 64].

Algae-bacteria interactions in the phycosphere are facilitated by proximity between cells which often takes the form of bacterial colonization of host algal cells. The flavobacterium *Croceibacter atlanticus* inhibits diatom growth when it attaches to the diatom, possibly in order to increase colonizable surface area of the diatom [65]. This interaction exemplifies antagonism of bacteria upon the microalgae. Here, microscopy, flow cytometry and genomics work can be augmented by introducing MS-based metabolomics work to characterize which molecules are exchanged between the flavobacterium and the diatoms leading to the growth inhibition.

An example of the use of omics techniques to characterize symbiosis is that *Sulfitobacter’s* production of indole-3-acetic acid (IAA) increases *Pseudo-nitzschia’s* cell division rate (Fig. 3c) [66]. Metabolomics and transcriptomics were used to characterize this signaling molecule’s role, linking two of the ocean’s microorganisms. Furthermore, this *Sulfitobacter* responds with an increased growth rate only when co-cultured with *Pseudo-nitzschia*, likely by taking up tryptophan produced by the diatom, establishing a symbiotic relationship promoting growth in both organisms through the exchange of small signaling molecules. Environmental concentrations of IAA are sufficient to induce an ecologically relevant response as observed in culture. The study identified bacteria as the IAA producer by identifying transcripts of genes involved in IAA biosynthesis in both the laboratory and the environment, and through measurement of IAA in axenic culture experiments. However, in the environment, it is possible that some IAA sources from terrestrial runoff of plant-derived DOM affects the aquatic microbial community as IAA is a common plant hormone and bacterial natural product [67].

The small molecule cobalamin (Vitamin B_12_) is critical in regulating microbial communities. For example, diatoms scavenge cobalamin from the environment rather than make it themselves, following principles of the “Black Queen Hypothesis” of gene-loss reductive evolution in organisms, creating microbial dependences (Fig. 3d) [68, 69]. The term is an analogy to the situation in the game “Hearts”, when a player strategizes to not to take the queen of spade cards [68]. Cobalamin availability controls which phytoplankton can grow well where. It is needed as a cofactor for enzymes, and some plankton have evolved enzymes (metE) that function without a cobalamin cofactor, though less efficiently. The model diatom *Thalassiosira pseudonana* was used to study how diatoms respond metabolically to cobalamin limitation for the first time through both targeted and non-targeted metabolomic methods [70]. Cobalamin-limitation to diatom *Thalassiosira pseudonana*, which does not contain the metE gene in its genome, leading to a requisite cobalamin requirement (auxotrophy), revealed a differential response in production of several metabolites. Key findings include less dimethylsulfoniopropionate (DMSP) and glycine betaine under cobalamin limitation which normally are used to balance osmotic pressure, and that the diatoms enzymatically transform hydroxy-cobalamin into ado-cobalamin using an enzyme conserved among diatoms. These findings demonstrate the value of applying metabolomics to ecologically relevant processes like vitamin limitation to shed light on physiological responses.

Bacterial presence can also help dinoflagellates with iron acquisition through siderophore production: *Marinobacter* produces the weak siderophore vibrioferrin and co-occurs with dinoflagellate *Gymnodinium*, which may benefit from iron-chelating siderophore presence in iron-depleted environments [71–73]. Vibrioferrin is one of only a few marine siderophores with a characterized structure [74–77].

### Small molecules mediate microalgae-grazer interactions

Interactions between harmful algae and grazers are exemplified by the interactions between dinoflagellate *Alexandrium catenella* and copepod *Acartia tonsa* [78]. While copepods graze on toxic *A. catenella* to gain energy, they suffer negative outcomes to their reproductive abilities.

An important step in the discovery of chemical cues that mediate the interaction between zooplankton and their prey was the discovery of taurolipid “copepodamides” (Fig. 3e) [79]. Copepodamides are produced by copepods and induce a 20-time increase in dinoflagellate *Alexandrium*’s saxitoxin toxin production. Different species of copepods combine an amide and a taurine to 8 unique isoprenoid fatty acids to form this set of signaling molecule lipids that can be detected both in lab culture experiments and in the environment [79]. These novel molecules also induce bioluminescence in *Lingulodinium* dinoflagellates which confers a competitive advantage in deterring predation [80]. In addition to affecting dinoflagellate toxin production, copepodamide presence affects diatoms as: increased domoic acid toxin production from *Pseudo-nitzschia* and a decrease in colony size in *Skeletonema* [81]. Environmental concentrations of these molecules are relevant to cause restructuring of ecosystem dynamics and mediate predator-prey interactions in nature. Ecological warfare between copepods and diatoms were further underscored by the discovery that copepods that feed on blooming diatoms have a low hatching success rate due to a set of three 10-carbon aldehyde molecules that stop embryonic development [82].

Dimethyl sulfide (DMS) is an important source of sulfur-containing aerosol compounds (sulfate, methane sulfonate, sulfuric acid) which cause water vapor condensation and thereby promote cloud formation (Fig. 3f) [83]. DMS therefore holds a critical role in the natural climate feedback loop. In addition to its role in regulating climate, DMS, produced by some species of blooming plankton, has been found to regulate algae-grazer interactions [84]. The enzyme DMSP lyase was studied in bloom-forming coccolithophore *Emeliania huxleyi* and diatom *Thalassiosira pseudonana* and surprisingly found that it enhances grazing rates and promotes growth in grazers like *Oxyrrhis marina*, revealing the key ecological impact of the “eat-me” signaling molecule DMS.

### Multi-omics empowers ecosystem-level systems biology studies

Studies that integrate multiple ‘omics techniques may yield a more complete systems biology level understanding of any organism [85]. For example, the Ingalls group integrated transcriptomics and metabolomics to show that taxonomy and metabolomics can be linked. The results enabled them to link organisms with different metabolites they produce and consume with respect to light oscillations. One finding was that the *Crocosphaera* cyanobacteria produced the osmolyte trehalose at the end of the light period to store energy for the night. Integrated metabarcoding and metabolome data was utilized for the study of the effects of copepod feeding on planktonic interactions in aquatic environments, allowing for the conclusion that copepod feeding preferences are related to the metabolic stage of the prey rather than prey abundance [86].

### Capturing human impacts as a fundamental part of aquatic ecosystems

While metabolomics tools enable us to capture a broad range of metabolites, they are also well suited to detect and annotate a wide range of anthropogenic molecules. For example, aquatic ecosystems contain numerous xenobiotics that have become a fundamental part of their chemotypes [87].

Human-caused pollution to urban rivers was studied by combining bacterial metagenomic amplicon sequencing with GC-MS to characterize differences between clean and contaminated parts of an urban river, to understand how the organisms’ metabolomes shift when exposed to pollution [88]. In polluted waters that contained fecal bacteria, there were higher levels of sugar alcohols and short-chain fatty acids, which can enhance biofilm formation.

The chemical impact of humans on aquatic ecosystems is exhibited by the recent characterization of a salmon-killing xenobiotic, causing “urban runoff mortality syndrome” [89]. Although it is not a natural product or related to microbial turnover, anthropogenic contaminants represent a paradigm shift to the field of chemical ecology. The toxic molecule *N*-(1,3-dimethylbutyl)-*N*′-phenyl-p-phenylenediamine (6PPD) from tire tread rubber from roadway runoff is abundant in many freshwater creeks that are the home of U.S. Pacific Northwest Coho Salmon. Abiotic transformation processes are involved to produce 6PPD-quinone, found to be abundant in the aquatic ecosystem and toxic to Coho Salmon. The structure of this compound was characterized using UHPLC-MS/MS and NMR following extensive bio-activity guided fractionation steps (Fig. 3g). This example of humans unintentionally interfering with the salmon life cycle is one of many stories of how human waste runoff into aquatic ecosystems affects native organisms.

Another more indirect human impact on aquatic ecosystems is the increased abundance of the compound aetokthonotoxin and related die-offs of bald eagle populations in the south of the US [90]. The discovery of “The Eagle Killer” molecule aetokthonotoxin typifies how small molecules, facilitated through anthropogenic impacts and produced at the base of the food web can affect the whole food chain. The *Aetokthonos* cyanobacteria colonizes a freshwater invasive plant *Hydrilla* and produces a molecule aetokthonotoxin which bioaccumulates up the food web to top predator bald eagles, causing neurological disease, and ultimately the death of eagles (Fig. 3h). As aetokthonotoxin contains multiple bromine atoms, it has been speculated that its biosynthesis benefits from increased bromide salt concentrations related to anthropogenic sources. The identification of aetokthonotoxin and its linkage to the decline of bald eagle populations was a milestone for ecotoxicologists, as the novel toxin can now be monitored and better regulated. For example, monitoring of bromide levels in reservoirs will aid in bald eagle conservation. The discovery of this unprecedented natural product was facilitated by atmospheric-pressure matrix assisted laser desorption/ionization MS imaging (AP-MALDI-MSI) which was used to study the cyanobacterial colonies on the plant’s leaves to detect small molecules produced by the bacteria in situ, and to identify the molecular formula of the novel molecule.

Finally, anthropogenic impacts significantly alter aquatic environments, for example through ocean warming and ocean acidification, which decreases calcification and causes bleaching events in corals [91, 92]. Nutrient loading in aquatic environments has been shown to increase biomass and calcification and carbon fixation rates of coral *Acropora pulchra* [93]. Multi-omics studies using transcriptomics, proteomics, and metabolomics have been used to characterize the metabolic exchange and regulation of the coral-microalgal symbiotic relationships. Some of the key findings include new insights into heat stress influencing protein folding, antioxidant biosynthetic gene expression, and catabolism of lipid stores in some symbioses [94–96]. As a result of detrimental anthropogenic impacts on the natural environment, humans used metabolomics as a method to aid in the conservation of endangered freshwater mussels, critical in their role as ecosystem engineers [97, 98]. To understand the higher mortality in mussels relocated to alternative environments or to captivity for management purposes, mussels were interrogated with non-targeted GC-MS and LC-MS [97]. It was found that relocation of mussels from the native environment changed amino acid and nucleotide metabolism, likely reflecting a stress response due to the change in habitat.

## Emerging metabolomics tools for aquatic chemical ecology

### New tools enable large-scale metabolomics data analysis and compound annotation

With the constant improvement of mass spectrometers’ sensitivity, scan speed and accessibility, thousands to millions of individual mass spectra are typically obtained per metabolomics study. Similar to sequencing-based omics studies, data analysis has thus become a major bottleneck and has reached a point that is far beyond manual data interpretation. Hence, scalable software solutions are indispensable to extract conclusions from data in metabolomics experiments. Fortunately, both mass spectrometer vendors and open-source developers have created numerous software tools for raw file processing, feature extraction, feature annotation, and downstream statistical analysis. Multiple software platforms, with graphical user interface (GUI), web-based apps, or scripting packages provide streamlined workflows that include quantification and enhanced detection of chromatographic features as well as annotation of MS data [99–106]. While each platform has unique capabilities and serves different user preferences, the interconnection of different platforms enables the best customizable data analysis solution, especially at the interface of feature detection, annotation and statistical analysis.

Feature-based Molecular Networking (FBMN) through the GNPS environment, for example, allows for the seamless import of processed raw mass spectrometry data, as well as export to further annotation and statistical analysis from a wide range of vendor and open source software tools [107–109]. FBMN builds upon molecular networking, which connects related molecules by their MS/MS spectral similarities [35]. FBMN can also make use of ion mobility separation information in addition to its algorithm to characterize chromatographic features and separate isomers with similar MS/MS spectra. The FBMN concept and workflow allows for improved annotation of mass spectra in combination with the ability for semi-quantitative or relative quantitative analysis, enhancing the user’s downstream research and potential for new compound discovery and quantification from understudied organisms such as from aquatic fungi and aquatic macroalgae [110].

Since “unknown” features in a non-targeted experiment typically represent the majority, additional tools are needed to bridge this large gap of unidentified data. In addition to matching experimental MS/MS spectra against spectral libraries, other tools predict MS/MS fragments from structure data and match those predicted MS/MS fragments against the experimental spectra. Such approaches are typically called in silico spectrum annotation. As structural databases are significantly larger than spectral libraries (typically 10-100 fold), in silico annotation tolls have much larger coverage, and allow for annotation of features at varying confidence and information levels (e.g. molecular formula, chemical class, or planar structures) [111, 112]. Recent advances include the use of Network Annotation Propagation (NAP), which was developed to improve the accuracy of existing in silico spectrum annotation tools by adding molecular networking to re-rank in silico annotations between connected nodes to generate consensus annotations within the networks, which typically improves the confidence in the annotations [111]. In recent years, several tools have been developed to improve in silico spectrum annotation further. SIRIUS is a computational tool that analyzes isotope patterns and fragmentation trees, which are related to the fragment peaks of a molecule to identify molecular formulas [113]. SIRIUS is often used in combination with CSI:Finger ID, which allows the matching of fragmentation trees of experimental spectra with fragmentation trees from structural databases. ZODIAC, improves the prediction of molecular formulas from SIRIUS by also taking into account similar fragments and losses between from other derivatives from spectral networks [114]. CANOPUS (class assignment and ontology prediction using MS) is another computational tool within the SIRIUS software suite, that allows chemical class level prediction through fragmentation trees [109]. An application of this tool on a crude extract of a cyanobacteria *Rivularia sp*. demonstrated these predictions by superclass, class and subclass: CANOPUS identified the major compound to be a cyclic peptide that was not annotated or identified by other computational methods. NMR was used to confirm the structural predictions and characterize the group of rivulariapeptolide, allowing for a greater understanding of aquatic natural products from microorganisms [115]. The easy-to-use GUI and integration of these tools improves the user experience for in silico spectrum annotation. Another way to predict unknown molecules is to improve the size of the library: “Suspect IDs” have been generated by using molecular networking and analog searches, in which unknown MS/MS spectra are matched against the GNPS MS/MS spectral library. Spectra that show high spectral similarity, but different precursor masses with a delta matching common modifications, were considered to be related compounds, which were then included in a new “suspect library” available on GNPS [116]. This generates new library spectra out of known library spectra, making the library of standards more comprehensive.

### Data sharing and computational advances allow the reutilization of existing metabolomics data

The increasing willingness of researchers to share their data in public repositories and the communities’ efforts to organize the data and their meta-data allows for global repository-scale analyses through new software tools such as the MS Search Tool (MASST) [100, 117]. Akin to BLAST which is commonly used to compare biological sequence information against public databases, MASST allows querying a single MS/MS spectrum, for example from a known compound, against all public MS/MS data. The output will tell in which dataset the spectrum or in a broader sense the compound of interest is present. This information can then be used to hypothesize about the occurrence or origin of certain compounds, even without the need for full identification.

 MASST has been used for example for the repository scale spectrum search of the potent neurotoxin domoic acid. Surprisingly, the results of this analysis indicated that domoic acid was present in data sets from coral reefs in Hawaii, an environment that is typically not known for the presence of *Pseudo-nitzschia*, the microalgae that produces domoic acid. In another study, MASST was used for the contextualization of metabolites and xenobiotics, detected in coastal environments [118].

Besides spectrum matching of single spectra against entire MS repositories through MASST, there are more tools emerging to perform multivariate analysis, such as Principal Coordinate Analysis (PCoA) of whole repositories [107]. An important aspect for repository scale analysis is the use and organization of controlled metadata. Through the ReDu template in GNPS for example, the user can provide a metadata sheet with controlled vocabulary in addition to their raw metabolomics data, which allows the seamless integration of their data into a global analysis, such as PCoA [119]. With the rise of LC-MS/MS centric meta-analysis tools and repositories, there has been significant effort from the imaging MS (IMS) community to centralize data storage which can then be leveraged for high-confidence annotation of ion features [120]. With the rise of new instrument platforms such as for single cell proteomics or µm-range lateral resolved MALDI imaging platforms, new and effective strategies are needed for the high demands of data analysis, clean-up and annotation [121, 122]. Data from the METASPACE imaging mass spectrometry repository has also been used to filter out background features from single-cell MALDI imaging data with a software tool called SpaceM [123]. Such tools further integrate other spatial data, such as fluorescent microscopy images, or single cell transcriptomics/ genomics data. Such data integration approaches are fundamental to increase for example spatial resolution (through image fusion) or compare transcriptional to translational regulation [121, 122]. In the microbial ecology context, multi-omics data layers, such as combined metabolomics and transcriptomics/ genomics data can further be used to identify biosynthesis genes or the producing organisms [100, 124, 125]. Connecting molecules to organisms is therefore not an easy to tackle problem, despite it being of high interest to many scientific fields [15, 23].

### Bioinformatic approaches to integrate metabolomes with community composition

The CoNet tool can be used to detect associations between genes, metabolites, and taxa and also hosts a command-line usage that allows for interactions between these variables to be easily visualized in Cytoscape [126]. This computational tool does co-occurrence analysis and network interference analysis to predict relationships between measurements of genes, metabolites, and taxa. It also can take metadata as an input to look for associations between environmental variables and other metadata variables with these biological measurements. CoNet was applied to determine which microorganisms (based on 16 S rRNA gene data) in Arctic soil are co-correlated and anti-correlated with pH and identify microorganisms that cross-feed in a complex environmental microbiological sample [126]. Another recent tool to connect metabolites with microorganisms is “mmvec” which uses neural networks to determine how likely a metabolite is to be linked with microorganisms [125]. In order to generate databases of these associations and to verify computationally predicted associations, characterizing metabolites of laboratory-cultured phytoplankton species and natural phytoplankton communities is paramount [127].

### Advances in functional annotation of metabolites at scale

While the identity of most metabolites, especially in complex environmental systems, are still unknown, even less information is available about the biological function and activities that these compounds might have. Thus, in addition to tackling the bottleneck of metabolite annotation, novel experimental strategies are needed to assign metabolite function at scale. The idea of pairing functional assays with high-throughput metabolomics, often referred to as functional metabolomics, is hence very compelling [128, 129]. While bioactivity-guided assays have been applied and refined in the natural product field for decades, the pairing to high-throughput metabolomics approaches is not straightforward [130–132]. Major bottlenecks are the scalability of bio-assays related to the fraction number per metabolomics sample, unambiguous assignment of activities to individual compounds as well as their structural annotation. Recent advances in the combination of bio-activity and non-targeted metabolomics workflows include the integration of high-throughput phenotyping and metabolomics as well as molecular networking approaches with bioactivity assays for the assignment of antibacterial and antiviral assays [133–135], as well as molecular interactions such as metabolite-metal and metabolite-protein binding [115, 136, 137]. Similar, bioactivity-guided LC-MS approaches are widely used in the environmental chemistry community, often termed effect-directed analysis [138, 139]. For example, connecting toxicity with non-targeted chemical profiling and downstream de novo structure elucidation led to the above mentioned discovery of a salmon mortality-inducing compound from rubber wear-off from car tires that enter aquatic systems through storm water run-off [89].

## Conclusions and future directions

Non-targeted metabolomics tools offer great potential to provide molecular insights into complex ecosystems and expand biological conclusions from multi-omics studies. The top four key advantages and emerging solutions that we see for this field are: (1) improved metabolite annotation and democratization of spectral libraries and data, (2) improved functional metabolite assignments (i.e. toxicity) (3) integration of multi-omics datasets, and (4) contextualization and re-use of data. Yet, key challenges that remain are: (1) Lack of medium-scope studies to bridge the knowledge gap between quantitative targeted and non-targeted qualitative environmental studies, (2) ultra-complexity of environmental samples, (3) and annotation of remaining unknown metabolites.

Inherent limitations of current metabolomics approaches are that targeted studies lose the scope of the thousands of unstudied molecules [140, 141], whereas, non-targeted studies face the challenge of limited library sizes that most often only allow the annotation of less than ten percent of the total number of features. The improvement of quantitative capabilities for non-targeted studies will pave the way forward to bridging this knowledge gap. While the challenge of spectral annotation through spectral libraries remains, it is constantly improved by the increase in spectral library coverage, in silico spectral annotation tools and compound suspect identifications [109, 113, 116].

Despite these potential advances, challenges that an unknown proportion of these spectra are from abiotic backgrounds, and another fraction are from the detection of isotopes that are not unique compounds will remain. Other challenges arise from the inherent ultra-complex nature of environmental samples: One study aimed to separate isomers in DOM with high performance liquid chromatography found that the ultra-complex nature of the mixture was a central barrier and that the complexity is an order of magnitude higher than what was previously expected [142]. Furthermore, highly hydrophilic biodegradable dissolved molecules are not well detected with typical methods of solid phase extraction to concentrate DOM prior to mass spectrometry, so many of these species are likely overlooked during DOM analysis [143]. New experimental methodology is still needed to improve the chemical extraction and characterization of environmental metabolomics samples, and to provide standardized and reproducible computational frameworks for the community to share, re-utilize, and contextualize data [144, 145]. In addition to limitations in aquatic metabolomics, a recent review of machine learning strategies for integrating multi-omic marine datasets identified some challenges identified with data integration to be the variability and noise of data, limited metadata, inaccessible computational tools, and high cost [146].

### Connecting chemotypes with genotypes

Along with advances in metagenomics and increased accessibility of high-quality Metagenome-Assembled Genome (MAG), important future developments in metabolomics tools for aquatic microbial ecology may include the better integration of MAGs and genome mining strategies with metabolomic data. New tools and infrastructures like NPOmix and The Paired Omics Data Platform pave the way to connect thereby molecules to their biosynthesis genes [124–126, 147]. It is estimated that two-thirds of the work on a multi-omic projects goes to data processing and integration, highlighting how the current data analysis approaches are a time and energy consuming processes, with much room for improvement [148].

### Metabolomics methods for global change assessment

We anticipate that metabolomics tools will play a central role in assessing the impact of climate change on aquatic systems. It has been predicted that climate change induced rising ocean temperatures favor larger and more frequent harmful cyanobacterial blooms [149], and understanding and perhaps leveraging chemical cues that influence harmful algal blooms [150], is of great importance. Metabolomics tools may play an integral part to identify such metabolites in multi-stressor experiments. An important aspect hereby well be the scaling from single chemical entities and interactions to chemotype-wide ecology of molecules. A fully structurally resolved inventory of all metabolites / DOM pool, would be the basis to build up models for DOM persistence (intrinsic and emergent) along with ecological/environmental parameters [15, 151, 152]. Multi-dimensional DOM fractionation and LC-MS/MS analysis, combined with bioactivity screening approaches, such as cytological profiling high content screening methods or antimicrobial assays, could link DOM chemical composition to biological functions. When done at high resolution and scale, identifying new natural products and their biological properties can be done directly from environmental samples [153]. Such developments will not only drastically improve our understanding of the DOM black box, but also unleash its tremendous molecular complexity as a potential source of structural and pharmaceutical/biotechnological novelty.

### Waiting for the “AlphaFold-Moment” in metabolomics

Machine learning, and in particular, advanced deep learning tools like AlphaFold, and large language models (LLMs), are transforming the field of biological research [154]. AlphaFold, developed by DeepMind, has made it possible to accurately predict the 3D structures of proteins, which has historically been a fundamental challenge in structural biology [155]. This breakthrough in machine learning has the potential to accelerate the discovery of new drugs and therapies, as well as help us better understand the underlying mechanisms of many diseases. Meanwhile, LLMs, like OpenAI’s GPT-4, are being used to analyze large amounts of biomedical literature, making it possible to quickly identify new hypotheses and connections between metabolites and organisms [156]. This can lead to new insights into the relationships between genes, proteins, metabolites and microbial community dynamics, offering completely new scales to describe and understand ecosystem function. A similar breakthrough in the field of proteomics occurred when the SEQUEST software was developed to match tandem mass spectra from peptides to database sequences [157].

Besides advanced AI tools such as ChatGTP, many machine learning approaches are already well established in the metabolomics community. Especially in the realm of supervised multivariate statistics, machine learning classification tools such as random forest and XG-Boost are commonly used [42, 158]. At the same time, some of the above mentioned in silico spectrum annotation tools make use of deep neural networks to learning MS/MS fragmentation, compound class prediction, or de novo structure elucidation and are rapidly improving [110, 114, 159]. However, high confidence compound annotation through the prediction of MS/MS spectra from structure libraries is still an unsolved problem, and the field is still waiting for an “AlphaFold Moment” in mass spectrometry-based metabolomics.

## References

[1] Azam F, Fenchel T, Field JG, Gray JS, Meyer-Reil LA, Thingstad F (1983). The ecological role of water-column microbes in the sea. Mar Ecol Prog Ser.

[2] Morris RM, Rappé MS, Connon SA, Vergin KL, Siebold WA, Carlson CA (2002). SAR11 clade dominates ocean surface bacterioplankton communities. Nature.

[3] Gannon F (2002). Molecular biology—what’s in a name?. EMBO Rep..

[4] Hasin Y, Seldin M, Lusis A (2017). Multi-omics approaches to disease. Genome Biol.

[5] Lloyd-Price J, Arze C, Ananthakrishnan AN, Schirmer M, Avila-Pacheco J, Poon TW (2019). Multi-omics of the gut microbial ecosystem in inflammatory bowel diseases. Nature.

[6] Pinu FR, Beale DJ, Paten AM, Kouremenos K, Swarup S, Schirra HJ (2019). Systems biology and multi-omics integration: Viewpoints from the metabolomics research community. Metabolites.

[7] Dumas T, Courant F, Fenet H, Gomez E (2022). Environmental metabolomics promises and achievements in the field of aquatic ecotoxicology: Viewed through the pharmaceutical lens. Metabolites.

[8] Pomfret SM, Brua RB, Izral NM, Yates AG (2020). Metabolomics for biomonitoring: an evaluation of the metabolome as an indicator of aquatic ecosystem health. Environ Rev.

[9] Viant MR (2008). Recent developments in environmental metabolomics. Mol Biosyst.

[10] Ribbenstedt A, Ziarrusta H, Benskin JP (2018). Development, characterization and comparisons of targeted and non-targeted metabolomics methods. PLOS ONE.

[11] Gorrochategui E, Jaumot J, Lacorte S, Tauler R (2016). Data analysis strategies for targeted and untargeted LC-MS metabolomic studies: Overview and workflow. TrAC Trends Anal Chem.

[12] Blunt JW, Carroll AR, Copp BR, Davis RA, Keyzers RA, Prinsep MR (2018). Marine natural products. Nat Prod Rep..

[13] Molinski TF, Dalisay DS, Lievens SL, Saludes JP (2009). Drug development from marine natural products. Nat Rev Drug Discov.

[14] da Silva RR, Dorrestein PC, Quinn RA (2015). Illuminating the dark matter in metabolomics. Proc Natl Acad Sci USA.

[15] Moran MA, Kujawinski EB, Schroer WF, Amin SA, Bates NR, Bertrand EM (2022). Microbial metabolites in the marine carbon cycle. Nat Microbiol.

[16] Belhaj MR, Lawler NG, Hoffman NJ (2021). Metabolomics and lipidomics: expanding the molecular landscape of exercise biology. Metabolites.

[17] Holm HC, Fredricks HF, Bent SM, Lowenstein DP, Ossolinski JE, Becker KW (2022). Global ocean lipidomes show a universal relationship between temperature and lipid unsaturation. Science.

[18] Venter JC, Adams MD, Myers EW, Li PW, Mural RJ, Sutton GG (2001). The sequence of the human genome. Science.

[19] Chekan JR, Fallon TR, Moore BS (2020). Biosynthesis of marine toxins. Curr Opin Chem Biol.

[20] Meyer S, Kehr J-C, Mainz A, Dehm D, Petras D, Süssmuth RD (2016). Biochemical dissection of the natural diversification of microcystin provides lessons for synthetic biology of NRPS. Cell Chem Biol.

[21] Gohlke RS (1959). Time-of-flight mass spectrometry and gas-liquid partition chromatography. Anal Chem.

[22] Holmes JC, Morrell FA (1957). Open access repository-scale propagated nearest neighbor suspect spectral library for untargeted metabolomics. Appl Spectrosc.

[23] Chadha S Plant–microbe interaction: gene-to-metabolite network. In: Jogaiah S, Abdelrahman M (eds). *Bioactive Molecules in Plant Defense: Signaling in Growth and Stress*. 2019. Springer International Publishing, Cham, pp 75–100.

[24] Aharoni A, Goodacre R, Fernie AR (2023). Plant and microbial sciences as key drivers in the development of metabolomics research. Proc Natl Acad Sci USA.

[25] Tolstikov VV, Lommen A, Nakanishi K, Tanaka N, Fiehn O (2003). Monolithic silica-based capillary reversed-phase liquid chromatography/electrospray mass spectrometry for plant metabolomics. Anal Chem.

[26] Plumb RS, Johnson KA, Rainville P, Smith BW, Wilson ID, Castro-Perez JM (2006). UPLC/MS(E); a new approach for generating molecular fragment information for biomarker structure elucidation. Rapid Commun Mass Spectrom RCM.

[27] Aharoni A, Ric de Vos CH, Verhoeven HA, Maliepaard CA, Kruppa G, Bino R (2002). Nontargeted metabolome analysis by use of fourier transform ion cyclotron mass spectrometry. Omics J Integr Biol.

[28] Swartz ME (2005). UPLC^TM^: an introduction and review. J Liq Chromatogr Relat Technol.

[29] Makarov A (2000). Electrostatic axially harmonic orbital trapping:  a high-performance technique of mass analysis. Anal Chem.

[30] Hardman M, Makarov AA (2003). Interfacing the orbitrap mass analyzer to an electrospray ion source. Anal Chem.

[31] Kopka J, Schauer N, Krueger S, Birkemeyer C, Usadel B, Bergmüller E (2005). Gmd@csb.db: the golm metabolome database. Bioinformatics.

[32] Montenegro-Burke JR, Guijas C, Siuzdak G (2020). Metlin: a tandem mass spectral library of standards. Methods Mol Biol Clifton NJ.

[33] Jarvie T (2005). Next generation sequencing technologies. Drug Discov Today Technol.

[34] Kaplan K, Graf S, Tanner C, Gonin M, Fuhrer K, Knochenmuss R (2010). Resistive glass im-tofms. Anal Chem.

[35] Wang Z, Gerstein M, Snyder M (2009). RNA-Seq: a revolutionary tool for transcriptomics. Nat Rev Genet.

[36] Perez de Souza L, Alseekh S, Scossa F, Fernie AR (2021). Ultra-high-performance liquid chromatography high-resolution mass spectrometry variants for metabolomics research. Nat Methods.

[37] Watrous J, Roach P, Alexandrov T, Heath BS, Yang JY, Kersten RD (2012). Marine metabolomics: a method for nontargeted measurement of metabolites in seawater by gas chromatography–mass spectrometry. Proc Natl Acad Sci USA.

[38] Jain M, Olsen HE, Paten B, Akeson M (2016). The oxford nanopore minion: delivery of nanopore sequencing to the genomics community. Genome Biol.

[39] Pomyen Y, Wanichthanarak K, Poungsombat P, Fahrmann J, Grapov D, Khoomrung S (2020). Deep metabolome: applications of deep learning in metabolomics. Comput Struct Biotechnol J.

[40] Sen P, Lamichhane S, Mathema VB, McGlinchey A, Dickens AM, Khoomrung S (2021). Deep learning meets metabolomics: a methodological perspective. Brief Bioinform.

[41] Petrick LM, Shomron N (2022). AI/ML-driven advances in untargeted metabolomics and exposomics for biomedical applications. Cell Rep. Phys Sci.

[42] Liebal UW, Phan ANT, Sudhakar M, Raman K, Blank LM (2020). Machine learning applications for mass spectrometry-based metabolomics. Metabolites.

[43] Zhang H, Meng G, Mao F, Li W, He Y, Gin KY-H (2019). Use of an integrated metabolomics platform for mechanistic investigations of three commonly used algaecides on cyanobacterium, *Microcystis aeruginosa*. J Hazard Mater.

[44] Via CW, Glukhov E, Costa S, Zimba PV, Moeller PDR, Gerwick WH (2018). The metabolome of a cyanobacterial bloom visualized by MS/MS-based molecular networking reveals new neurotoxic smenamide analogs (C, D, and E). Front Chem.

[45] Brunson JK, McKinnie SMK, Chekan JR, McCrow JP, Miles ZD, Bertrand EM (2018). Biosynthesis of the neurotoxin domoic acid in a bloom-forming diatom. Science.

[46] Fiorini F, Borgonuovo C, Ferrante MI, Brönstrup M (2020). A metabolomics exploration of the sexual phase in the marine diatom *Pseudo-nitzschia multistriata*. Mar Drugs.

[47] Koester I, Quinlan ZA, Nothias L-F, White ME, Rabines A, Petras D (2022). Illuminating the dark metabolome of *Pseudo-nitzschia*-microbiome associations. Environ Microbiol.

[48] Poulson-Ellestad KL, Jones CM, Roy J, Viant MR, Fernandez FM, Kubanek J (2014). Metabolomics and proteomics reveal impacts of chemically mediated competition on marine plankton. Proc Natl Acad Sci USA.

[49] Poulin RX, Hogan S, Poulson-Ellestad KL, Brown E, Fernández FM, Kubanek J (2018). *Karenia brevis* allelopathy compromises the lipidome, membrane integrity, and photosynthesis of competitors. Sci Rep..

[50] Poulin RX, Poulson-Ellestad KL, Roy JS, Kubanek J (2018). Variable allelopathy among phytoplankton reflected in red tide metabolome. Harmful Algae.

[51] Hassanpour B, Blair N, Aristilde L (2022). Metabolomics analysis of unresolved molecular variability in stoichiometry dynamics of a stream dissolved organic matter. Water Res.

[52] Garayburu-Caruso VA, Danczak RE, Stegen JC, Renteria L, Mccall M, Goldman AE (2020). Using community science to reveal the global chemogeography of river metabolomes. Metabolites.

[53] Seymour JR, Amin SA, Raina J-B, Stocker R (2017). Zooming in on the phycosphere: the ecological interface for phytoplankton–bacteria relationships. Nat Microbiol.

[54] Cirri E, Pohnert G (2019). Algae−bacteria interactions that balance the planktonic microbiome. N Phytol.

[55] Giovannoni SJ, Tripp HJ, Givan S, Podar M, Vergin KL, Baptista D (2005). Genome streamlining in a cosmopolitan oceanic bacterium. Science.

[56] Newton RJ, Jones SE, Eiler A, McMahon KD, Bertilsson S (2011). A guide to the natural history of freshwater lake bacteria. Microbiol Mol Biol Rev MMBR.

[57] Cai H, Jiang H, Krumholz LR, Yang Z (2014). Bacterial Community composition of size-fractioned aggregates within the phycosphere of cyanobacterial blooms in a eutrophic freshwater lake. PLOS ONE.

[58] Garcia SL, Nuy JK, Mehrshad M, Hampel JJ, Sedano-Nuñez VT, Buck M, et al. Taxonomic and functional diversity of aquatic heterotrophs is sustained by dissolved organic matter chemodiversity. bioRxiv. 2022. 10.1101/2022.03.21.485019.

[59] Giovannoni SJ, Cameron Thrash J, Temperton B (2014). Implications of streamlining theory for microbial ecology. ISME J.

[60] Shibl AA, Isaac A, Ochsenkühn MA, Cárdenas A, Fei C, Behringer G (2020). Diatom modulation of select bacteria through use of two unique secondary metabolites. Proc Natl Acad Sci USA.

[61] Seyedsayamdost MR, Carr G, Kolter R, Clardy J (2011). Roseobacticides: small molecule modulators of an algal-bacterial symbiosis. J Am Chem Soc.

[62] Seyedsayamdost MR, Case RJ, Kolter R, Clardy J (2011). The jekyll-and-hyde chemistry of *Phaeobacter gallaeciensis*. Nat Chem.

[63] Espiñeira JM, Novo Uzal E, Gómez Ros LV, Carrión JS, Merino F, Ros Barceló A (2011). Distribution of lignin monomers and the evolution of lignification among lower plants. Plant Biol Stuttg Ger.

[64] Martone PT, Estevez JM, Lu F, Ruel K, Denny MW, Somerville C (2009). Discovery of lignin in seaweed reveals convergent evolution of cell-wall architecture. Curr Biol.

[65] van Tol HM, Amin SA, Armbrust EV (2017). Ubiquitous marine bacterium inhibits diatom cell division. ISME J.

[66] Amin SA, Hmelo LR, van Tol HM, Durham BP, Carlson LT, Heal KR (2015). Interaction and signaling between a cosmopolitan phytoplankton and associated bacteria. Nature.

[67] Spaepen S, Vanderleyden J (2011). Auxin and plant-microbe interactions. Cold Spring Harb Perspect Biol.

[68] Morris JJ, Lenski RE, Zinser ER. The black queen hypothesis: evolution of dependencies through adaptive gene loss. mBio. 3: e00036-12. 10.1128/mBio.00036-12 PMC3315703 22448042

[69] Giovannoni SJ (2012). Vitamins in the sea. Proc Natl Acad Sci USA.

[70] Heal KR, Kellogg NA, Carlson LT, Lionheart RM, Ingalls AE (2019). Metabolic consequences of cobalamin scarcity in the diatom *Thalassiosira pseudonana* as revealed through metabolomics. Protist.

[71] Amin SA, Green DH, Küpper FC, Carrano CJ (2009). Vibrioferrin, an unusual marine siderophore: iron binding, photochemistry, and biological implications. Inorg Chem.

[72] Yamamoto S, Fujita Y, Okujo N, Minami C, Matsuura S, Shinoda S (1992). Isolation and partial characterization of a compound with siderophore activity from *Vibrio parahaemolyticus*. FEMS Microbiol Lett.

[73] Amin SA, Küpper FC, Green DH, Harris WR, Carrano CJ (2007). Boron binding by a siderophore isolated from marine bacteria associated with the toxic dinoflagellate *Gymnodinium catenatum*. J Am Chem Soc.

[74] Martinez JS, Zhang GP, Holt PD, Jung H-T, Carrano CJ, Haygood MG (2000). Self-Assembling Amphiphilic Siderophores from Marine Bacteria. Science.

[75] Martinez JS, Haygood MG, Butler A (2001). Identification of a natural desferrioxamine siderophore produced by a marine bacterium. Limnol Oceanogr.

[76] Martinez JS, Carter-Franklin JN, Mann EL, Martin JD, Haygood MG, Butler A (2003). Structure and membrane affinity of a suite of amphiphilic siderophores produced by a marine bacterium. Proc Natl Acad Sci USA.

[77] Reid RT, Livet DH, Faulkner DJ, Butler A (1993). A siderophore from a marine bacterium with an exceptional ferric ion affinity constant. Nature.

[78] Abdulhussain AH, Cook KB, Turner AD, Lewis AM, Bibby TS, Mayor DJ (2021). The influence of the toxin-producing dinoflagellate, *Alexandrium catenella* (1119/27), on the survival and reproduction of the marine copepod, *Acartia tonsa*, during prolonged exposure. Front Mar Sci.

[79] Selander E, Kubanek J, Hamberg M, Andersson MX, Cervin G, Pavia H (2015). Predator lipids induce paralytic shellfish toxins in bloom-forming algae. Proc Natl Acad Sci USA.

[80] Prevett A, Lindström J, Xu J, Karlson B, Selander E (2019). Grazer-induced bioluminescence gives dinoflagellates a competitive edge. Curr Biol.

[81] Selander E, Berglund EC, Engström P, Berggren F, Eklund J, Harðardóttir S (2019). Copepods drive large-scale trait-mediated effects in marine plankton. Sci Adv.

[82] Miralto A, Barone G, Romano G, Poulet SA, Ianora A, Russo GL (1999). The insidious effect of diatoms on copepod reproduction. Nature.

[83] Charlson RJ, Lovelock JE, Andreae MO, Warren SG (1987). Oceanic phytoplankton, atmospheric sulphur, cloud albedo and climate. Nature.

[84] Shemi A, Alcolombri U, Schatz D, Farstey V, Vincent F, Rotkopf R (2021). Dimethyl sulfide mediates microbial predator–prey interactions between zooplankton and algae in the ocean. Nat Microbiol.

[85] Boysen AK, Carlson LT, Durham BP, Groussman RD, Aylward FO, Ribalet F (2016). Particulate metabolites and transcripts reflect diel oscillations of microbial activity in the surface ocean. mSystems.

[86] Ray JL, Althammer J, Skaar KS, Simonelli P, Larsen A, Stoecker D (2016). Metabarcoding and metabolome analyses of copepod grazing reveal feeding preference and linkage to metabolite classes in dynamic microbial plankton communities. Mol Ecol.

[87] Cancelada L, Torres RR, Garrafa Luna J, Dorrestein PC, Aluwihare LI, Prather KA (2022). Assessment of styrene-divinylbenzene polymer (PPL) solid-phase extraction and non-targeted tandem mass spectrometry for the analysis of xenobiotics in seawater. Limnol Oceanogr Methods.

[88] Beale DJ, Karpe AV, Ahmed W, Cook S, Morrison PD, Staley C (2017). A community multi-omics approach towards the assessment of surface water quality in an urban river system. Int J Environ Res Public Health.

[89] Tian Z, Zhao H, Peter KT, Gonzalez M, Wetzel J, Wu C (2021). A ubiquitous tire rubber–derived chemical induces acute mortality in coho salmon. Science.

[90] Breinlinger S, Phillips TJ, Haram BN, Mareš J, Martínez Yerena JA, Hrouzek P (2021). Hunting the eagle killer: a cyanobacterial neurotoxin causes vacuolar myelinopathy. Science.

[91] Hughes TP, Kerry JT, Álvarez-Noriega M, Álvarez-Romero JG, Anderson KD, Baird AH (2017). Global warming and recurrent mass bleaching of corals. Nature.

[92] Hughes TP, Barnes ML, Bellwood DR, Cinner JE, Cumming GS, Jackson JBC (2017). Coral reefs in the anthropocene. Nature.

[93] Tanaka Y, Miyajima T, Koike I, Hayashibara T, Ogawa H (2007). Imbalanced coral growth between organic tissue and carbonate skeleton caused by nutrient enrichment. Limnol Oceanogr.

[94] Petrou K, Nunn BL, Padula MP, Miller DJ, Nielsen DA (2021). Broad scale proteomic analysis of heat-destabilised symbiosis in the hard coral *Acropora millepora*. Sci Rep..

[95] Cziesielski MJ, Liew YJ, Cui G, Schmidt-Roach S, Campana S, Marondedze C (2018). Multi-omics analysis of thermal stress response in a zooxanthellate cnidarian reveals the importance of associating with thermotolerant symbionts. Proc Biol Sci.

[96] Hillyer KE, Dias DA, Lutz A, Roessner U, Davy SK (2017). Mapping carbon fate during bleaching in a model cnidarian symbiosis: the application of 13C metabolomics. N Phytol.

[97] Roznere I, Watters GT, Wolfe BA, Daly M (2017). Effects of relocation on metabolic profiles of freshwater mussels: Metabolomics as a tool for improving conservation techniques. Aquat Conserv Mar Freshw Ecosyst.

[98] Waller D, Putnam J, Steiner JN, Fisher B, Burcham GN, Oliver J (2023). Targeted metabolomics characterizes metabolite occurrence and variability in stable freshwater mussel populations. Conserv Physiol.

[99] Weber RJM, Lawson TN, Salek RM, Ebbels TMD, Glen RC, Goodacre R (2016). Computational tools and workflows in metabolomics: an international survey highlights the opportunity for harmonisation through Galaxy. Metabolomics.

[100] Wang M, Carver JJ, Phelan VV, Sanchez LM, Garg N, Peng Y (2016). Sharing and community curation of mass spectrometry data with GNPS. Nat Biotechnol.

[101] Tautenhahn R, Patti GJ, Rinehart D, Siuzdak G (2012). XCMS online: a web-based platform to process untargeted metabolomic data. Anal Chem.

[102] Helf MJ, Fox BW, Artyukhin AB, Zhang YK, Schroeder FC (2022). Comparative metabolomics with Metaboseek reveals functions of a conserved fat metabolism pathway in *C. elegans*. Nat Commun.

[103] Tsugawa H, Cajka T, Kind T, Ma Y, Higgins B, Ikeda K (2015). MS-DIAL: data-independent MS/MS deconvolution for comprehensive metabolome analysis. Nat Methods.

[104] Pluskal T, Castillo S, Villar-Briones A, Orešič M (2010). MZmine 2: Modular framework for processing, visualizing, and analyzing mass spectrometry-based molecular profile data. BMC Bioinforma.

[105] Rainer J, Vicini A, Salzer L, Stanstrup J, Badia JM, Neumann S (2022). A modular and expandable ecosystem for metabolomics data annotation in R. Metabolites.

[106] Schmid R, Heuckeroth S, Korf A, Smirnov A, Myers O, Dyrlund TS (2023). Integrative analysis of multimodal mass spectrometry data in MZmine 3. Nat Biotechnol.

[107] Bolyen E, Rideout JR, Dillon MR, Bokulich NA, Abnet CC, Al-Ghalith GA (2019). Reproducible, interactive, scalable and extensible microbiome data science using QIIME 2. Nat Biotechnol.

[108] Nothias L-F, Petras D, Schmid R, Dührkop K, Rainer J, Sarvepalli A (2020). Feature-based molecular networking in the GNPS analysis environment. Nat Methods.

[109] Dührkop K, Nothias L-F, Fleischauer M, Reher R, Ludwig M, Hoffmann MA (2021). Systematic classification of unknown metabolites using high-resolution fragmentation mass spectra. Nat Biotechnol.

[110] Fan B, Grauso L, Li F, Scarpato S, Mangoni A, Tasdemir D (2022). Application of feature-based molecular networking for comparative metabolomics and targeted isolation of stereoisomers from algicolous fungi. Mar Drugs.

[111] Silva RR, da, Wang M, Nothias L-F, Hooft JJJ, van der, Caraballo-Rodríguez AM, Fox E (2018). Propagating annotations of molecular networks using in silico fragmentation. PLOS Comput Biol.

[112] Krettler CA, Thallinger GG (2021). A map of mass spectrometry-based in silico fragmentation prediction and compound identification in metabolomics. Brief Bioinform.

[113] Dührkop K, Fleischauer M, Ludwig M, Aksenov AA, Melnik AV, Meusel M (2019). SIRIUS 4: a rapid tool for turning tandem mass spectra into metabolite structure information. Nat Methods.

[114] Ludwig M, Nothias L-F, Dührkop K, Koester I, Fleischauer M, Hoffmann MA (2020). Database-independent molecular formula annotation using Gibbs sampling through ZODIAC. Nat Mach Intell.

[115] Reher R, Aron AT, Fajtová P, Stincone P, Wagner B, Pérez-Lorente AI (2022). Native metabolomics identifies the rivulariapeptolide family of protease inhibitors. Nat Commun.

[116] Bittremieux W, Avalon NE, Thomas SP, Kakhkhorov SA, Aksenov AA, Gomes PWP, et al. Open access repository-scale propagated nearest neighbor suspect spectral library for untargeted metabolomics. bioRxiv. 2022. 10.1101/2022.05.15.490691. 10.1038/s41467-023-44035-y PMC10733301 38123557

[117] Wang M, Jarmusch AK, Vargas F, Aksenov AA, Gauglitz JM, Weldon K (2020). Mass spectrometry searches using MASST. Nat Biotechnol.

[118] Petras D, Minich JJ, Cancelada LB, Torres RR, Kunselman E, Wang M (2021). Non-targeted tandem mass spectrometry enables the visualization of organic matter chemotype shifts in coastal seawater. Chemosphere.

[119] Jarmusch AK, Wang M, Aceves CM, Advani RS, Aguirre S, Aksenov AA (2020). ReDU: a framework to find and reanalyze public mass spectrometry data. Nat Methods.

[120] Alexandrov T, Ovchinnikova K, Palmer A, Kovalev V, Tarasov A, Stuart L, et al. METASPACE: A community-populated knowledge base of spatial metabolomes in health and disease. bioRxiv 2019. 539478. 10.1101/539478.

[121] Brunner A-D, Thielert M, Vasilopoulou C, Ammar C, Coscia F, Mund A (2022). Ultra-high sensitivity mass spectrometry quantifies single-cell proteome changes upon perturbation. Mol Syst Biol.

[122] Kompauer M, Heiles S, Spengler B (2017). Atmospheric pressure MALDI mass spectrometry imaging of tissues and cells at 1.4-μm lateral resolution. Nat Methods.

[123] Rappez L, Stadler M, Triana S, Gathungu RM, Ovchinnikova K, Phapale P (2021). SpaceM reveals metabolic states of single cells. Nat Methods.

[124] Schorn MA, Verhoeven S, Ridder L, Huber F, Acharya DD, Aksenov AA (2021). A community resource for paired genomic and metabolomic data mining. Nat Chem Biol.

[125] Morton JT, Aksenov AA, Nothias LF, Foulds JR, Quinn RA, Badri MH (2019). Learning representations of microbe–metabolite interactions. Nat Methods.

[126] Faust K, Raes J (2016). CoNet app: inference of biological association networks using Cytoscape. F1000Research.

[127] Heal KR, Durham BP, Boysen AK, Carlson LT, Qin W, Ribalet F (2021). Marine community metabolomes carry fingerprints of phytoplankton community composition. mSystems.

[128] Yang R, Fredman G, Krishnamoorthy S, Agrawal N, Irimia D, Piomelli D (2011). Decoding functional metabolomics with docosahexaenoyl ethanolamide (DHEA) identifies novel bioactive signals. J Biol Chem.

[129] Peng B, Li H, Peng X-X (2015). Functional metabolomics: from biomarker discovery to metabolome reprogramming. Protein Cell.

[130] Baumgartner B, Erdelmeier CAJ, Wright AD, Rali T, Sticher O (1990). An antimicrobial alkaloid from *Ficus septica*. Phytochemistry.

[131] Kersten RD, Lane AL, Nett M, Richter TKS, Duggan BM, Dorrestein PC (2013). Bioactivity-Guided Genome Mining Reveals the Lomaiviticin Biosynthetic Gene Cluster in *Salinispora tropica*. ChemBioChem.

[132] Shinichi Sakemi, Toshio Ichiba, Shigeo Kohmoto, Gabriel Saucy (1988). Higa Tatsuo. Isolation and structure elucidation of onnamide A, a new bioactive metabolite of a marine sponge, *Theonella sp*. J Am Chem Soc.

[133] Ochoa JL, Bray WM, Lokey RS, Linington RG (2015). Phenotype-guided natural products discovery using cytological profiling. J Nat Prod.

[134] Kurita KL, Linington RG (2015). Connecting phenotype and chemotype: high-content discovery strategies for natural products research. J Nat Prod.

[135] von Eckardstein L, Petras D, Dang T, Cociancich S, Sabri S, Grätz S (2017). Total synthesis and biological assessment of novel albicidins discovered by mass spectrometric networking. Chem – Eur J.

[136] Aron AT, Petras D, Schmid R, Gauglitz JM, Büttel I, Antelo L (2022). Native mass spectrometry-based metabolomics identifies metal-binding compounds. Nat Chem.

[137] Behnsen J, Zhi H, Aron AT, Subramanian V, Santus W, Lee MH (2021). Siderophore-mediated zinc acquisition enhances enterobacterial colonization of the inflamed gut. Nat Commun.

[138] Nothias L-F, Nothias-Esposito M, da Silva R, Wang M, Protsyuk I, Zhang Z (2018). Bioactivity-based molecular networking for the discovery of drug leads in natural product bioassay-guided fractionation. J Nat Prod.

[139] Brack W (2003). Effect-directed analysis: a promising tool for the identification of organic toxicants in complex mixtures?. Anal Bioanal Chem.

[140] Nowinski B, Moran MA (2021). Niche dimensions of a marine bacterium are identified using invasion studies in coastal seawater. Nat Microbiol.

[141] Olofsson M, Ferrer-González FX, Uchimiya M, Schreier JE, Holderman NR, Smith CB (2022). Growth-stage-related shifts in diatom endometabolome composition set the stage for bacterial heterotrophy. ISME Commun.

[142] Hawkes JA, Patriarca C, Sjöberg PJR, Tranvik LJ, Bergquist J (2018). Extreme isomeric complexity of dissolved organic matter found across aquatic environments: Extreme isomeric complexity of DOM. Limnol Oceanogr Lett.

[143] Grasset C, Groeneveld M, Tranvik LJ, Robertson LP, Hawkes JA. Hydrophilic species are the most biodegradable components of freshwater dissolved organic matter. Environ Sci Technol. 2023. 10.1021/acs.est.3c02175 PMC10501193 37646447

[144] Petras D, Koester I, Da Silva R, Stephens BM, Haas AF, Nelson CE (2017). High-resolution liquid chromatography tandem mass spectrometry enables large scale molecular characterization of dissolved organic matter. Front Mar Sci.

[145] Sogin EM, Puskás E, Dubilier N, Liebeke M (2019). Marine metabolomics: a method for nontargeted measurement of metabolites in seawater by gas chromatography–mass spectrometry. mSystems.

[146] Manochkumar J, Cherukuri AK, Kumar RS, Almansour AL, Ramamoorthy S, Efferth T (2023). A critical review of machine-learning for “multi-omics” marine metabolite datasets. Comput Biol Med.

[147] Leão TF, Wang M, da Silva R, Gurevich A, Bauermeister A, Gomes PWP (2022). NPOmix: a machine learning classifier to connect mass spectrometry fragmentation data to biosynthetic gene clusters. PNAS Nexus.

[148] Palsson B, Zengler K (2010). The challenges of integrating multi-omic data sets. Nat Chem Biol.

[149] Paerl HW, Huisman J (2008). Blooms like it hot. Science.

[150] Hennon GMM, Dyhrman ST (2020). Progress and promise of omics for predicting the impacts of climate change on harmful algal blooms. Harmful Algae.

[151] Dittmar T, Lennartz ST, Buck-Wiese H, Hansell DA, Santinelli C, Vanni C (2021). Enigmatic persistence of dissolved organic matter in the ocean. Nat Rev Earth Environ.

[152] Lambidis S, Schramm T, Steuer-Lodd K, Farrell S, Stincone P, Schmid R, et al. Two-Dimensional Liquid Chromatography Tandem-Mass Spectrometry Untangles the Deep Metabolome of Marine Dissolved Organic Matter. ChemRxiv. 2023. 10.26434/chemrxiv-2023-j1bxh. 10.1021/acs.est.4c07173 39413296

[153] Bogdanov A, Salib MN, Chase AB, Hammerlindl H, Muskat MN, Luedtke S, et al. Small molecule in situ resin capture-an organism independent strategy for natural product discovery. bioRxiv 2023; 2023–03. 10.1101/2023.03.02.530684.

[154] Jumper J, Evans R, Pritzel A, Green T, Figurnov M, Ronneberger O (2021). Highly accurate protein structure prediction with AlphaFold. Nature.

[155] Dill KA, Ozkan SB, Shell MS, Weikl TR (2008). The protein folding problem. Annu Rev Biophys.

[156] Floridi L, Chiriatti M (2020). Gpt-3: its nature, scope, limits, and consequences. Minds Mach.

[157] James P Proteome research: mass spectrometry. 2001. Springer, Berlin, Heidelberg.

[158] Chen T, Guestrin C Xgboost: a scalable tree boosting system. *Proc. 22nd ACM SIGKDD Int. Conf. Knowl. Discov. Data Min*. 2016. Association for Computing Machinery, New York, NY, USA, pp 785-94.

[159] Stravs MA, Dührkop K, Böcker S, Zamboni N (2022). MSNovelist: de novo structure generation from mass spectra. Nat Methods.

[160] Aksenov AA, da Silva R, Knight R, Lopes NP, Dorrestein PC (2017). Global chemical analysis of biology by mass spectrometry. Nat Rev Chem.

