# Publisher Correction: Progress and challenges in exploring aquatic microbial communities using non-targeted metabolomics

**DOI:** 10.1038/s41396-023-01552-4

**Published:** 2023-11-22

**Authors:** Monica Thukral, Andrew E. Allen, Daniel Petras

**Affiliations:** 1 University of California San Diego, Scripps Institution of Oceanography, La Jolla, CA USA; 2 J. Craig Venter Institute, Microbial and Environmental Genomics Group, La Jolla, CA USA; 3 University of Tuebingen, CMFI Cluster of Excellence, Tuebingen, Germany; 4 Department of Biochemistry, University of California Riverside, Riverside, CA USA

**Keywords:** Microbial ecology, Microbial ecology

Correction to: *The ISME Journal* 10.1038/s41396-023-01532-8, published online 19 October 2023

Unfortunately, there was a mix-up in the numbering of references and reference titles during typesetting between the proofs and the published article.

The moved references have been listed below and the correct reference numbers and corrected titles for two references are provided.

· Page 3

o Figure 2 Caption

§ [46, 109, 115] --> [47,109,115]

§ [46] --> [47]

· Page 4

o Table 1

§ [63] -->[61]

§ [72] --> [73]

§ [91] -->[89]

· Reference Section

o The title of  reference [68] has been corrected.

§ Previous:

· Morris JJ, Lenski RE, Zinser ER. The application of nanopore sequencing tech- nology to the study of dinoflagellates: a proof of concept study for rapid sequence-based discrimination of potentially harmful algae. mBio. 3: e00036-12.

§ Corrected:

· Morris JJ, Lenski RE, Zinser ER. The Black Queen Hypothesis: Evolution of Dependencies through Adaptive Gene Loss. mBio. 3: e00036-12.

o The title of reference [96] has been corrected.

§ Previous:

· Hillyer KE, Dias DA, Lutz A, Roessner U, Davy SK. Machine learning applications for mass spectrometry-based metabolomics. N Phytol. 2017;214:1551–62.

§ Corrected:

· Hillyer KE, Dias DA, Lutz A, Roessner U, Davy SK. Mapping carbon fate during bleaching in a model cnidarian symbiosis: the application of 13C metabolomics. N Phytol. 2017;214:1551–62.

The original article has been corrected.

